# αvβ3-targeted gas vesicles for ultrasound molecular imaging of tumors

**DOI:** 10.3389/fbioe.2026.1808539

**Published:** 2026-03-27

**Authors:** Xiong Shen, Hanlin Li, Tingting Liu, Yuanyuan Wang, Chenxing Liu, Yao Chen, Maosen Hong, Yusheng Lin, Zhijie Guo, Zhenzhou Li, Fei Yan

**Affiliations:** 1 Graduate School, Guangxi University of Chinese Medicine, Nanning, China; 2 Department of Ultrasound, The second People’s Hospital of Shenzhen, The First Affiliated Hospital of Shenzhen University, Shenzhen, China; 3 State Key Laboratory of Quantitative Synthetic Biology, Shenzhen Institute of Synthetic Biology, Shenzhen Institutes of Advanced Technology, Chinese Academy of Sciences, Shenzhen, China; 4 School of Clinical Medicine, Clinical College, Liaoning He’s Medical University, Liaoning, China; 5 Department of Pharmacy, Baishui Town Central Hospital, Yueyang, China; 6 Department of Vascular Surgery, The Second People’s Hospital of Shenzhen, The First Affiliated Hospital of Shenzhen University, Shenzhen, China; 7 Department of Ultrasound, Shenzhen Bao’an Chinese Medicine Hospital, Guangzhou University of Chinese Medicine, Shenzhen, China

**Keywords:** gas vesicles, tumors, ultrasound contrast agents, ultrasound molecular imaging, αvβ3 integrin

## Abstract

**Purpose:**

To achieve ultrasound molecular imaging (UMI) of various tumor types, this study developed a novel integrin-targeted probe, RGD-hGVs, based on gas vesicles (GVs) derived from *Halobacterium salinarum* NRC-1 (*Halo*) and cyclic RGDfK peptides. The application potentials of UMI were evaluated in osteosarcoma and melanoma.

**Materials and Methods:**

RGD-hGV acoustic nanoprobes were constructed by conjugating Halo-derived GVs with cyclic RGD peptides. Morphology was characterized by PCM and TEM, and hydrodynamic size and zeta potential were measured. *In vitro* targeting to bEnd.3, K7M2, and B16-F10 cells was evaluated by flow cytometry and confocal microscopy. Ultrasound imaging was performed in osteosarcoma and melanoma models, followed by immunofluorescence staining for αvβ3 and probe distribution. Biocompatibility was assessed by hemolysis, CCK-8 assays, serum biochemistry, and H&E staining of major organs.

**Results:**

RGD-hGVs (∼240 nm) demonstrated targeted binding to tumor cells (bEnd.3, K7M2, B16-F10) *in vitro* and *in vivo*. They produced stronger, longer-lasting tumor ultrasound signals than non-targeted controls, with immunofluorescence confirming localization to tumor vasculature and cells, and showed no significant toxicity.

**Conclusion:**

RGD-hGVs may serve as a novel UMI probe, suitable for diagnosing multiple tumors.

## Graphical Abstract

**Figure:**
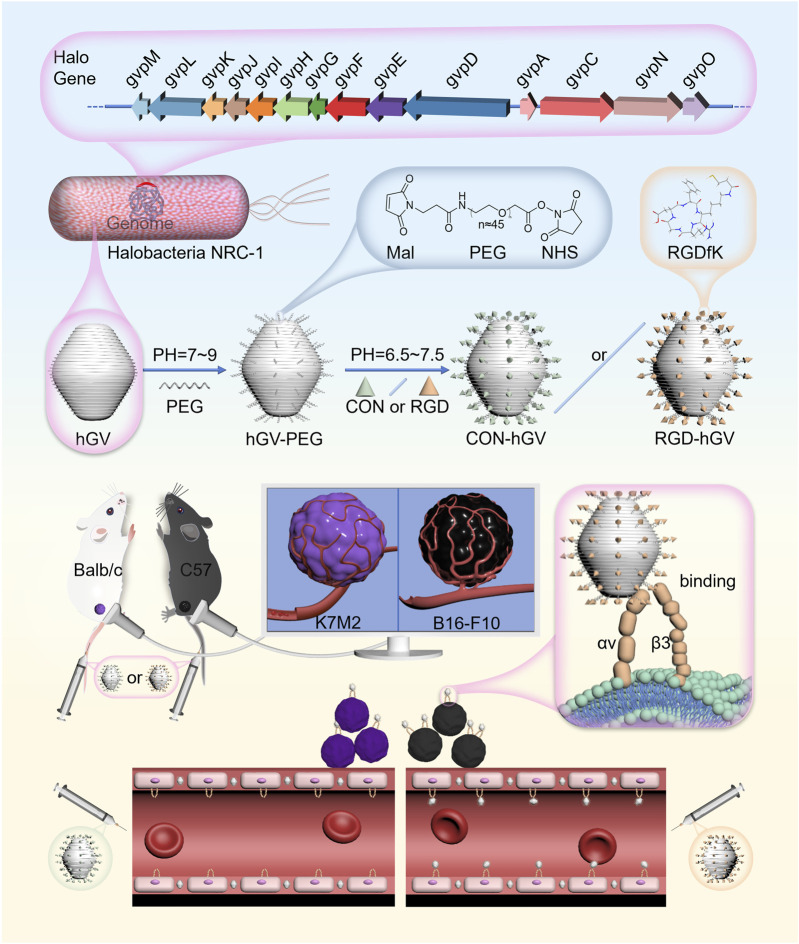

## 1 Introduction

Osteosarcoma and melanoma are both highly malignant solid tumors characterized by rapid progression and a tendency to metastasize distantly at an early stage. Once these tumors reach an advanced stage or recurrence with metastasis, the prognosis is generally poor (Evdokimova et al., 2022; Leiter et al., 2020; Zhu et al., 2023). Despite advances in surgery, chemotherapy, immunotherapy, and targeted therapy, a considerable number of patients are either diagnosed at an intermediate to advanced stage or experience recurrence and metastasis following treatment (Arnold et al., 2022). A major contributing factor is the limited sensitivity of current imaging techniques, including CT and MRI, for detecting early-stage micrometastases (Sun et al., 2022). Furthermore, these techniques are unable to dynamically reflect molecular events, such as tumor angiogenesis and invasive metastasis. All of these limitations hinder timely and accurate guidance for precise risk stratification and treatment efficacy assessment. Therefore, the development of imaging technologies capable of *in vivo*, real-time, non-invasive assessment of tumor molecular features holds significant importance for early diagnosis, risk stratification, and treatment monitoring (Bai et al., 2023).

UMI has advanced rapidly in recent years. By conjugating specific ligands to the surface of ultrasound contrast agents (UCAs), molecular probes can specifically bind to disease-related molecular targets, enabling visualization of tumor-associated molecular markers (Jang et al., 2023). Moreover, UMI preserves the inherent advantages of conventional ultrasound, such as real-time imaging, no radiation, point-of-care availability, and cost-effectiveness. However, clinical contrast agents such as SonoVue and Sonazoid are phospholipid microbubbles (1–5 µm), which largely restricts their efficient extravasation into the tumor stroma for direct detection. Furthermore, their short circulation time and rapid signal decay often require repeated administration. As a result, microbubble-based acoustic molecular probes are hindered in the tumor application. In recent years, biosynthetic GVs have garnered attention as nanoscale UCAs (Ling et al., 2024; Ling et al., 2023; Shen et al., 2024). These naturally gas-filled protein nanostructures, produced by certain bacteria and archaea, typically with 100–200 nm in hydrodynamic particle size. They are encoded by Gvp gene clusters and form rigid protein shells approximately 2 nm thick. Compared to chemically synthesized nanobubbles, gas vesicles offer advantages such as genetic programmability, a relatively straightforward and scalable preparation process and excellent biocompatibility (Wang et al., 2024).

In our earlier work, we successfully biosynthesized GVs derived from Halo (Zhu et al., 2023; Gong et al., 2022). These GVs combine favorable acoustic properties, good biocompatibility, and vascular permeability, highlighting their promise as nanoscale contrast agents (Yu et al., 2025; Wang et al., 2025a). In this work, RGD-hGVs were constructed as a targeted contrast agent through Michael addition–mediated conjugation of RGD-targeting peptide onto the gas vesicle surface. Furthermore, we evaluated the molecular imaging performance of this contrast agent in enabling early detection of osteosarcoma and melanoma.

## 2 Materials and methods

### 2.1 Materials

The targeted peptide RGD, cyclo[RGDfK(Mpa)], and the control peptide Con, cys-MEHGRWG, were obtained from Shanghai Apeptide Co., Ltd. and GL Biochemical (Shanghai) Co., Ltd., respectively. Reagents and materials included maleimide–poly(ethylene glycol) 2000–NHS ester (MAL-PEG2000-NHS; CAS R-1106-2K; RISI Bio), dialysis bags (CAS YA-1068; SolarBio; Shenzhen Shangxing Technology Co., Ltd.), and hyaluronic acid (CAS 9067-32-7; MeilunBio; Dalian Meilun Biotechnology Co., Ltd.). The K7M2 and B16-F10 cell lines, as well as mouse bEnd.3 endothelial cells, were obtained from ATCC. BALB/c and C57 mice (approximately 20 g) were supplied by the Guangdong Provincial Medical Laboratory Animal Center. The CCK-8 reagent (CAS BS350B; BioSharp) was procured from Guangzhou Saige Biotechnology Co., Ltd.

### 2.2 Separation of GVs

The Halo culture was propagated in ATCC medium containing a trace-element supplement (100 mL total: 0.66 g ZnSO_4_·7H_2_O, 0.17 g MnSO_4_, 0.41 g ferrous ammonium sulfate, and 0.07 g CuSO_4_·5H_2_O) at 37 °C and 200 rpm for 7–10 days. The culture was subsequently poured into a separatory funnel and allowed to stand without agitation for 1–2 weeks, during which buoyant GV-bearing cells accumulated in the upper phase. The buoyant top fraction was collected and subjected to osmotic lysis in TMC buffer (1 L: 1.21 g Tris, 0.51 g MgCl_2_, and 0.29 g CaCl_2_·2H_2_O; pH 7.5). The resulting lysate was centrifuged at 300 *g* (4 °C, 3 h); this low-speed spin was repeated 3–5 rounds to isolate the GVs. GVs were produced in more than 10 independent batches, each defined as an independent culture followed by an independent purification. Unless otherwise stated, experiments were repeated using GVs from at least three different batches. To minimize batch-related confounding, GVs from different batches were normalized to the same OD_500_ before use, and the same imaging and analysis settings were applied across groups. GV concentration was determined by recording absorbance at a wavelength of 500 nm (OD_500_) with a UV–Vis microplate reader (Multiskan Go; Thermo Fisher Scientific, Waltham, MA, United States). In addition, PCM (Olympus IX83 inverted microscope, Tokyo, Japan) was used to observe hGVs and bacterial preparations, and purified hGVs were further assessed via TEM (Hitachi H-7500, Hitachi Ltd., Tokyo, Japan). Briefly, the bacterial cells and hGVs were adjusted by dilution to an appropriate density, spotted on coverslips precoated with 1% agarose, and imaged on an IX83-SIM PCM using a ×100 oil-immersion objective. For TEM, diluted hGVs were applied to copper grids, negatively stained with 2% phosphotungstic acid, air-dried at room temperature, and examined to assess hGV morphology.

### 2.3 Preparation and characterization of RGD-modified hGVs and control hGVs

First, 20 mg of Mal-PEG2000-NHS was incubated with 2 mL of hGVs (OD_500_ = 3.0) overnight at 4 °C with gentle mixing. Unreacted Mal-PEG2000-NHS was then eliminated via dialyzing against PBS (SolarBio; MWCO 1000 kDa). Next, RGD peptides were incubated with Mal-PEG2000-modified hGVs overnight at 4 °C to obtain targeted RGD-hGVs. The non-targeted control hGVs (Con-hGVs) were prepared following the same procedure. FITC-labeled GVs were obtained by incubating with FITC-tagged peptides, either FITC-RGD or FITC-Con. Free peptides were removed by dialysis. Fluorescence intensity was assessed using a microplate reader to confirm successful conjugation. The particle size and zeta potential of hGVs were measured with a Zetasizer analyzer (Zetasizer Nano S90, Malvern; Worcestershire, UK). The colloidal stability of PEG-hGVs in PBS and mouse serum was evaluated by monitoring hydrodynamic diameter over 6 days (DLS); no obvious size change was observed (Supplementary Figures S2A,B). hGVs (including functionalized hGVs) were stored in PBS (pH 7.4) at 4 °C until use. Stability characterization for RGD-hGVs during storage is provided in the Supplementary Material (Supplementary Figures S2C,D). All functionalized hGVs were processed under aseptic conditions in a laminar-flow clean bench and were sterile-filtered through a 0.22 µm membrane before use in downstream experiments.

### 2.4 *In vivo* imaging

A customized 1.0% (w/v) agarose gel was prepared as an *in vitro* imaging phantom. Subsequently, 20 μL of hGVs, Con-hGVs, or RGD-hGVs at different concentrations (optical density at 500 nm, OD_500_ = 2.0, 2.5, 3.0) and 180 µL of 1% hyaluronic acid were dispensed into the agarose gel wells. Ultrasound imaging was carried out on an ultrasound diagnostic system (Resona 9T, Mindray; Shenzhen, China) fitted with a L11-3U linear array transducer. The probe was placed in direct contact with the agarose gel surface, with the acoustic power set to 5.13%, contrast gain to 70 dB, and mechanical index (MI) to 0.145. A complete list of ultrasound imaging parameters is provided in the Supplementary Material (Supplementary Table S1). The imaging performance of hGVs was subsequently evaluated.

### 2.5 Cell culture

Mouse bEnd.3 endothelial cells, B16-F10 melanoma cells, and K7M2 mouse osteosarcoma cells were maintained in DMEM supplemented with 15% fetal bovine serum (Gibco) plus 1% penicillin–streptomycin. Cultures were incubated at 37 °C in a humidified incubator with 5% CO2.

### 2.6 *In vitro* cell targeting research

To evaluate cell-surface binding *in vitro*, bEnd.3 cells (∼80% confluence) were used. Cells (1 × 10^5^) were collected, suspended in 400 μL PBS, and distributed into 2 mL microcentrifuge tubes. FITC-RGD or FITC-Con (5 μg) was introduced into each tube, followed by incubation on ice for 10 min. For the competitive binding assay, free RGD peptide (5 μg) was pre-incubated with the cells for ∼20 min, after which 5 μg of FITC-RGD peptide was added and incubated for another 10 min. The suspensions were then washed 3 times with sterile PBS. K7M2 and B16-F10 cells were processed in an identical manner.

To test *in vitro* binding of RGD-hGVs, approximately (1 × 10^4^) bEnd.3 cells were plated in 24-well plates and cultured overnight to permit attachment, after which they were fixed with 4% paraformaldehyde. Each well received 100 μL of either FITC-RGD-hGVs or FITC-Con-hGVs (OD_500_ = 1.5). All samples were kept at room temperature in the dark for 10 min, to evaluate the *in vitro* association of RGD-hGVs with tumor cells, cells were plated, in 24-well plates and left overnight for attachment, and subsequently fixed in 4% paraformaldehyde, followed by three 5-min washes with cold PBS. After DAPI staining and washing, cell morphology was observed using a confocal microscope (A1R, Nikon; Japan). For competitive inhibition assays, unlabeled free RGD peptides were preincubated with cells at room temperature for 20 min before adding FITC-RGD-hGVs. The same cell adhesion assay procedure was performed for K7M2 and B16-F10 cells. Confocal microscopy was used to acquire the images, and mean fluorescence intensity ratio was quantified using ImageJ. All confocal images were acquired under identical settings across groups (Supplementary Table S2).

### 2.7 Animal model

Animal studies were conducted in accordance with applicable regulations and institutional policies for laboratory animal care and use. All imaging procedures were performed under sevoflurane anesthesia, and every effort was made to minimize animal distress. Inject 100 μL of PBS, containing K7M2 mouse osteosarcoma cells, into the right dorsal region of BALB/c mice. Similarly, inject 100 μL of PBS, containing B16-F10 mouse melanoma cells, into the left dorsal region of C57BL/6 mice. Tumor-bearing mice were used for ultrasound imaging once tumors became palpable (approximately 5–12 mm in diameter) following inoculation.

### 2.8 *In Vivo* ultrasound imaging detection

During ultrasound imaging, BALB/c mice were maintained under anesthesia on a heated pad with 1% isoflurane in oxygen (2 L/min). Five mice were randomly assigned to receive separate tail vein injections of either RGD-hGVs or Con-hGVs, each at a volume of 100 μL GV at 500 nm (OD_500_) value of 3.0. To minimize potential bias between injections in the same mouse, injections were spaced at least 30 min apart. Ultrasound imaging was conducted using a Mindray Resona 9T system. The parameters for *in vivo* imaging are the same as those for previous *in vitro* imaging, and images were acquired continuously for 10 min C57BL/6 mice were imaged using the same methodology. Ultrasound imaging was performed by multiple trained operators. To minimize inter-operator variability, all operators followed a standardized imaging protocol with fixed instrument settings (frequency, MI, gain, depth and dynamic range) and a consistent sample positioning/ROI analysis workflow. Contrast enhancement was quantified by measuring the average acoustic signal intensity within manually defined regions of interest (ROI) per frame using ImageJ.

### 2.9 Histological examination

Immediately after ultrasound imaging, animals were euthanized, and tumors were harvested and snap-frozen. Cryosections were cut on a cryostat (CM1950, Leica Microsystems, Heidelberg, Germany) and immunostained with an anti-β3 monoclonal antibody (GB112253-100; Servicebio). K7M2 and B16-F10 cells were immunolabeled with an anti-mouse β3 antibody. In addition, vessels were immunostained for CD31 according to the manufacturer’s instructions. Tissue fluorescence was examined using an upright fluorescence microscope (Nikon Eclipse C1; Japan).

### 2.10 Biosafety testing

Hemolysis was evaluated using freshly collected mouse blood. Briefly, 1 mL of blood from BALB/c mice was mixed with 2 mL PBS. After centrifugation (4,500 rpm, 5 min), the serum was removed and the erythrocyte pellet was retained. The pellet was washed 5 times with PBS and brought to 10 mL with PBS. RBC aliquots (250 μL) were then incubated with 1 mL PBS (negative control), 1 mL Triton X-100 (positive control), or 1 mL RGD-hGVs at different concentrations (OD_500_ = 0.5, 1.0, 1.5, 2.0, 2.5, and 3.0) at 37 °C for 3 h. After incubation, the supernatant was collected and centrifuged at 300 *g* for 20 min. Absorbance of 200 μL supernatant was measured at 541 nm using a microplate reader (Multiskan Go; Thermo Scientific, Waltham, MA, United States) to calculate the hemolysis rate. For cytotoxicity assessment, a CCK-8 assay was performed to quantify the effects of RGD-hGVs. B16-F10, K7M2, and bEnd.3 cells were seeded at (1 × 104) cells per well in 96-well plates and cultured for 24 h. RGD-hGV solutions with optical density at 500 nm (OD_500_) values of 0.5, 1.0, 1.5, 2.0, 2.5, and 3.0, corresponding to different concentrations, were dispensed into each well and incubated at 37 °C for 6 h. Then, 10 μL of CCK-8 reagent was added per well and incubated for a further 2–3 h. Cell viability was determined by measuring absorbance at 450 nm.

For *in vivo* biosafety evaluation, nine healthy mice were intravenously administered PBS, RGD-hGVs, or Con-hGVs (100 μL; OD_500_ = 3.0). After 7 days, blood was collected via the ophthalmic artery to assess liver function (alanine aminotransferase [ALT] and aspartate aminotransferase [AST]) and renal function (blood urea nitrogen [BUN] and creatinine [CREA]). Major organs (heart, liver, spleen, lung, and kidney) were then harvested, fixed in 4% (w/v) paraformaldehyde, and processed for hematoxylin and eosin (H&E) staining (Wuhan Savier Biotechnology Co., Ltd., China). Additionally, in a separate biosafety experiment, healthy mice were intravenously administered PBS or RGD-hGVs (100 μL; OD_500_ = 3.0). Complete blood count (CBC) was measured before injection and on day 3 post-injection, including white blood cell count (WBC) with granulocytes, red blood cell count (RBC), and hemoglobin (HGB) (Supplementary Figure S3).

### 2.11 Statistical analysis

Data are presented as mean ± SD. Comparisons between two groups were conducted using an unpaired t-test. For multiple-group comparisons, one-way analysis of variance (ANOVA) was used, followed by Bonferroni’s *post hoc* multiple-comparison test. Image processing and statistical analyses were performed using GraphPad Prism. A value of P < 0.05 was considered statistically significant; ** indicates P < 0.01; *** indicates P < 0.001; **** indicates P < 0.0001.

## 3 Results

### 3.1 Preparation and characterization of RGD-modified hGVs and control hGVs

**FIGURE 1:**
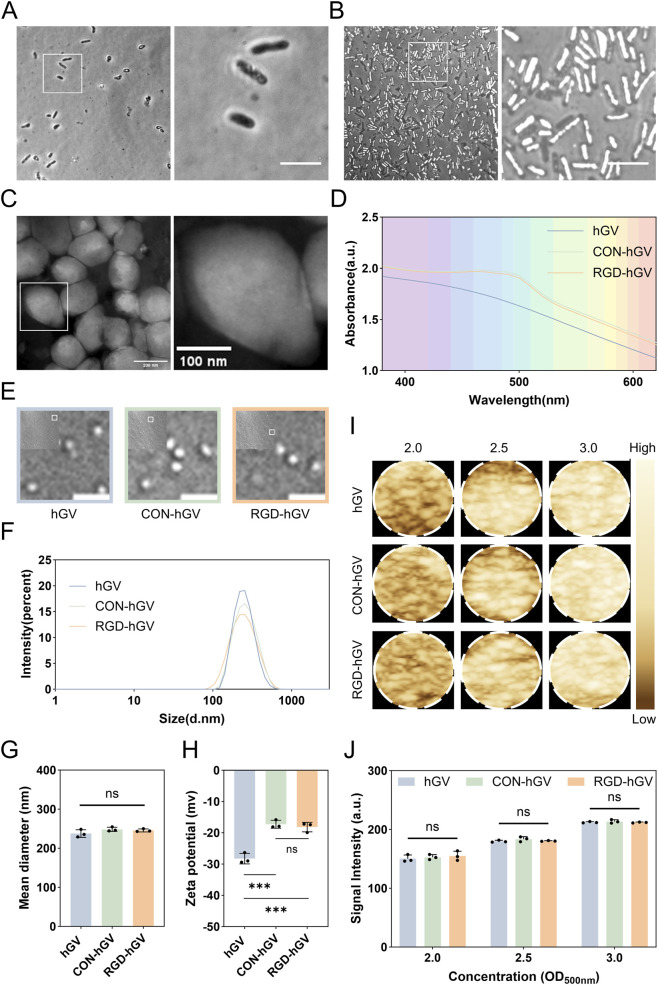
Characterization of hGVs, Con-hGVs and RGD-hGVs. PCM images of cultivated Halo on day 1 **(A)** and day 10 **(B)**. Scale bar: 5 μm. **(C)** TEM image of hGVs. Scale bar: 100 nm. **(D,E)** Absorbance spectra and PCM images of hGVs, Con-hGVs and RGD-hGVs. Scale bar: 1 μm. **(F–H)** Size distribution, the mean diameters and Zeta potential of hGVs, Con-hGVs and RGD-hGVs. **(I,J)** The representative ultrasound contrast images and the quantitative analysis of hGVs, Con-hGVs and RGD-hGVs at various concentrations (n = 3). *** for P < 0.001.

The production of hGV in Halo were observed via PCM on days 1 and 10 of cultivation. On day 1, hGVs appeared as punctate structures within the bacteria (Figure 1A), whereas by day 10, hGVs occupied nearly the entire bacterial cell volume (Figure 1B). Transmission electron microscopy revealed that hGVs exhibited a relatively uniform spindle-shaped morphology (Figure 1C). After purifying hGVs, Mal-PEG2000-hGV was prepared by incubation with Mal-PEG2000-NHS. Targeted hGVs (RGD-hGVs) were then synthesized via a Michael reaction, as shown in Supplementary Figures S2A,B. Figure 1D shows that FITC-labeled RGD or FITC-labeled control peptides were successfully conjugated to the surface of GVs, as evidenced by the characteristic FITC absorbance peak at 488 nm. Moreover, hGVs, RGD-hGVs, and control hGVs (Con-hGVs) displayed relatively uniform morphology, with particle sizes of 237.7 ± 5.6 nm, 245.8 ± 2.2 nm, and 248.5 ± 3.2 nm, respectively (Figures 1E–G). The zeta potentials of hGVs, RGD-hGVs, and Con-hGVs were −28.2 ± 1.0 mV, −18.2 ± 0.9 mV, and −17.3 ± 0.7 mV, respectively (Figure 1H). Both RGD and Con peptide functionalization significantly reduced the magnitude of the negative zeta potential compared with hGVs, whereas no significant difference was observed between RGD-hGVs and Con-hGVs (p > 0.05). This reduction is likely due to peptide decoration partially masking/neutralizing the native negatively charged surface groups.

### 3.2 hGVs *in vitro* imaging capability

The acoustic contrast imaging performance of hGVs was evaluated using *in vitro* ultrasound imaging. Imaging was conducted under the same ultrasound contrast parameters for hGVs and Con-hGVs at concentrations from OD_500_ = 2.0 to 3.0. Along with the increase of GV concentrations within this range of RGD-hGVs and Con-hGVs, the stronger contrast signals could obtained. Mean signal intensity was quantitatively evaluated, showing that Con-hGVs and RGD-hGVs produced similar contrast signals at identical concentrations, comparable to unmodified hGVs (Figures 1I,J).

### 3.3 *In vitro* cell targeting research

**FIGURE 2:**
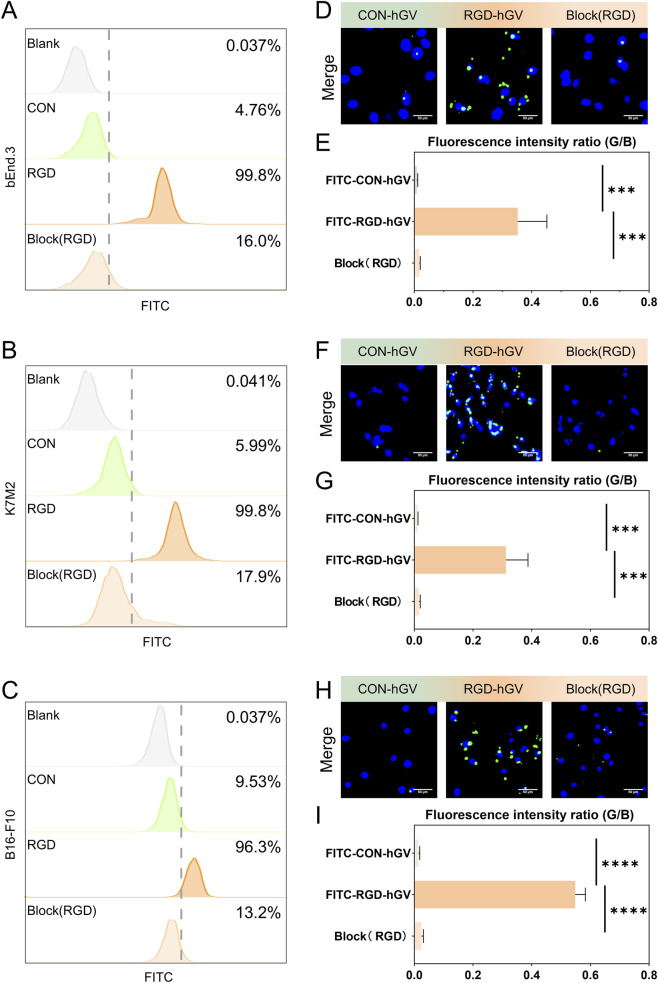
*In vitro* cell targeting studies. **(A–C)** Flow cytometry assay of bEnd.3 cells, K7M2 cells and B16-F10 cells incubated with FITC-Con, FITC-RGD or free RGD + FITC-RGD, respectively. Representative fluorescent microscope images of bEnd.3 cells **(D)**, K7M2 cells **(F)** and B16-F10 cells **(H)** incubated with FITC-Con-hGVs, FITC-RGD-hGVs or free RGD + FITC-RGD-hGVs. Green stands for FITC, and blue for cell nuclei stained with DAPI. Scale bar: 50 µm. **(E,G,I)** represent the quantitative analysis of the fluorescence intensity in **(D,F,H)**, respectively. *** for P < 0.001; **** for P < 0.0001.

Flow cytometric analysis demonstrated that the FITC-RGD peptide could effectively bind to bEnd.3 cells, K7M2 cells, and B16-F10 cells, whereas the control peptide did not bind to these cells. Quantitative analysis revealed that 99.8% of bEnd.3 cells, 99.8% of K7M2 cells, and 96.3% of B16-F10 cells showed strong fluorescence after exposure to FITC-RGD. In contrast, the fluorescence ratios in bEnd.3 and K7M2 cells treated with the control peptide were 5% and 6%, respectively. Notably, preincubation with RGD peptide significantly reduced FITC-RGD binding efficiency (Figures 2A–C). The full stepwise gating workflow (FSC/SSC debris exclusion, singlet gating using FSC-A vs. FSC-H, and definition of the FITC-positive threshold) and representative plots for each cell type are provided in the Supplementary Material (Supplementary Figures S4–S6).

Next, RGD-hGV was incubated with a monolayer of bEnd.3 cells in a 24-well plate to assess its binding efficiency to these cells. Figure 2D showed numerous RGD-hGVs associated with bEnd.3 cells. In contrast, only minimal binding of the non-targeted Con-hGV was observed. As anticipated, a small amount of RGD-hGVs was detected on bEnd.3 cells pre-incubated with free RGD peptide (Figure 2E). Similar results were observed with K7M2 and B16-F10 cells (Figures 2F–I). These findings indicated that RGD-hGVs bind to both bEnd.3 and K7M2 cells, as well as B16-F10 cells.

### 3.4 *In vivo* tumor imaging

**FIGURE 3:**
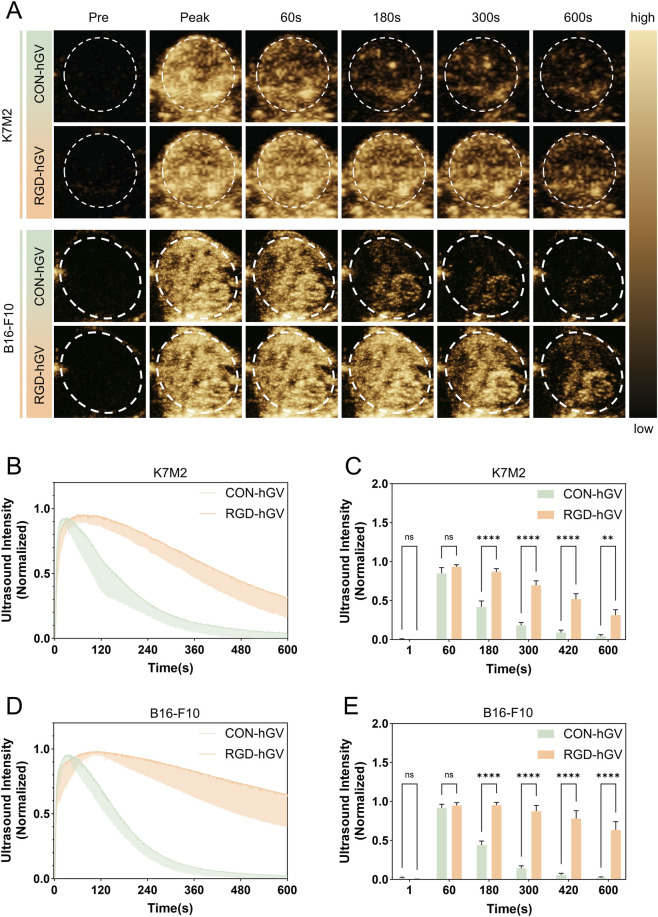
*In vivo* ultrasound molecular imaging of tumors. **(A)** Nonlinear contrast images of Con-hGVs and RGD-hGVs were obtained at various time points after intravenous injection in K7M2 osteosarcoma and B16-F10 melanoma tumor-bearing mice. Time–intensity curves of Con-hGVs and RGD-hGVs after intravenous injection in K7M2 osteosarcoma–bearing mice **(B)**, and corresponding tumor signal intensities at 1, 3, 5, 7, and 10 min post-injection **(C)**. Time–intensity curves of Con-hGVs and RGD-hGVs after intravenous injection in B16-F10 tumor–bearing mice **(D)**, and corresponding tumor signal intensities at 1, 3, 5, 7, and 10 min post-injection **(E)**.

Moreover, the *in vivo* imaging capability of RGD-hGVs was assessed in tumor-bearing mice. Con-hGVs and RGD-hGVs were administered via tail-vein injection in a randomized sequence. As shown in Figure 3A, the peak signal intensities in K7M2 and B16-F10 tumors were comparable between the Con-hGV and RGD-hGV groups (129.71 ± 5.18 A.U. vs. 125.81 ± 6.50 A.U.), with no statistically significant difference. In contrast, longitudinal analysis showed that tumor-associated signals in the RGD-hGV group remained consistently higher than those in the Con-hGV group, and the separation between groups became especially evident beyond 3 min. Figures 3B,C clearly demonstrated that the signal decay of RGD-hGVs in K7M2 osteosarcoma was slower than that of Con-hGVs. At 3, 5, 7, and 10 min post-injection, the ultrasound contrast signal intensities of RGD-hGVs were 2.27-, 3.80-, 4.79-, and 5.21-fold higher than those of Con-hGVs, respectively, confirming the superior UMI performance of RGD-hGVs in mice bearing tumors. Similarly, differences were also observed in B16-F10 tumors, with more pronounced contrast signal differences at 3, 5, 7, and 10 min post-injection. RGD-hGVs exhibited 2.54, 6.03, 9.21, and 11.20 times higher ultrasound contrast signals than those of Con-hGVs (Figures 3D,E). This indicated higher expression levels of the target receptor for RGD receptors in melanoma.

### 3.5 Tumor immunofluorescence staining

**FIGURE 4:**
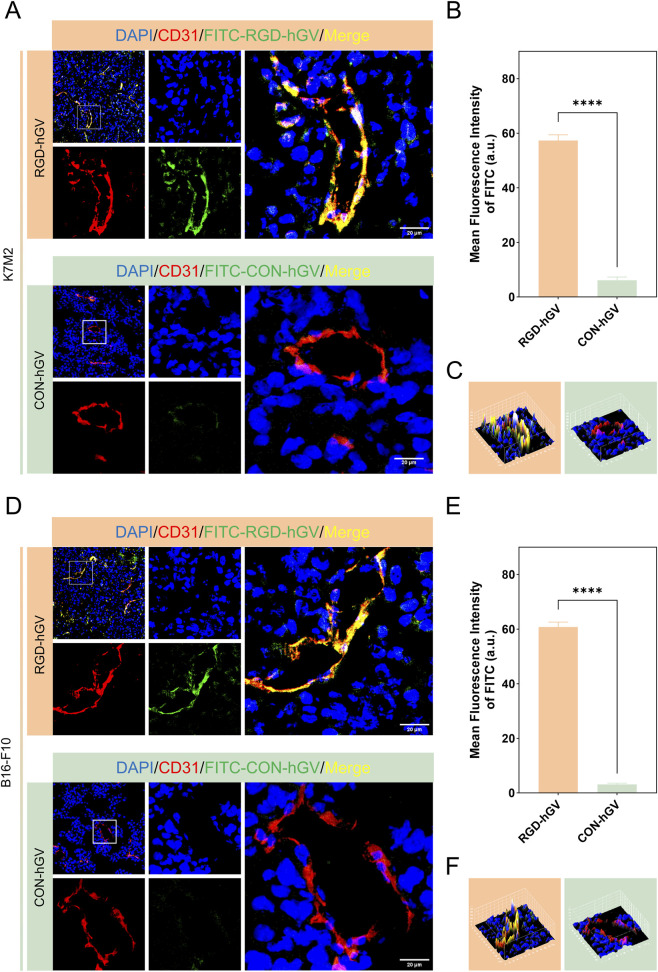
Immunofluorescence and quantitative analysis of tumors. **(A,B)** Vascular immunofluorescence images and their quantification were obtained 600 s following the intravenous injection of FITC-labeled Con-hGVs or RGD-hGVs in K7M2 osteosarcoma-bearing mice. **(C)** The corresponding 3D surface plot graphs are presented in. **(D,E)** Vascular immunofluorescence images and quantification were acquired 600 s following the intravenous injection of FITC-labeled Con-hGVs or RGD-hGVs in B16-F10 melanoma-bearing mice, with the corresponding 3D surface plot graphs displayed in **(F)**.

We have previously shown that αvβ3 integrin is expressed on angiogenic endothelial cells in K7M2 tumors. Consistently, the current study verified αvβ3 integrin expression on angiogenic endothelial cells in B16-F10 tumors (Supplementary Figures S2A,B). The 3D surface plot offered a more intuitive visualization of αvβ3 integrin expression on endothelial cells (Supplementary Figures S2C,D); it revealed strong colocalization with CD31 and tumor cells (Supplementary Figures S2E,F). To further validate the imaging differences in signal presence between RGD-hGVs and Con-hGVs within tumors, tumor tissues were harvested 10 min after injection of FITC-RGD-hGVs and FITC-Con-hGVs. Probe distribution was then analyzed via immunofluorescence. Results showed that the FITC-RGD-hGVs probe co-localized with CD31 and tumor cells in both osteosarcoma and melanoma tissues. This result explains the prolonged imaging signal of the targeted probe compared to the control, as demonstrated by the 3D Surface Plot (Figures 4A–F).

### 3.6 Biosafety testing

**FIGURE 5:**
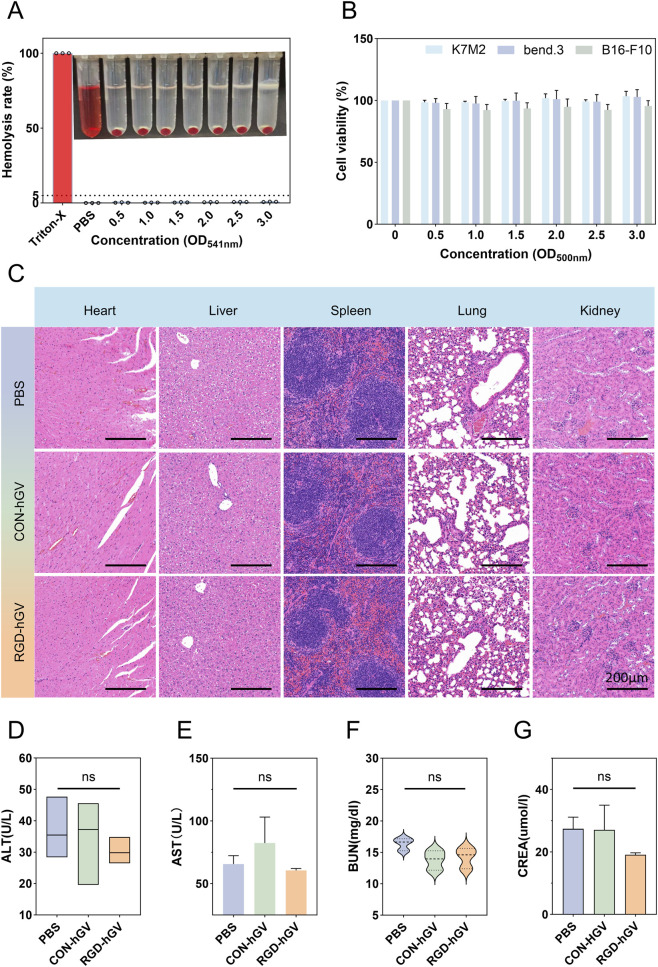
Biosafety analysis. **(A)** Hemolysis testing of RGD-hGVs at OD_500_ = 0.5, 1.0, 1.5, 2.0, 2.5, or 3.0, using Triton X-100 as the positive control and PBS as the negative control (n = 3). **(B)** Viability of bEnd.3, K7M2, and B16-F10 cells following exposure to RGD-hGVs at OD_500_ = 0.5, 1.0, 1.5, 2.0, 2.5, or 3.0 for 6 h (n = 3). **(C)** H&E histology of major organs from mice administered Con-hGVs (OD_500_ = 3.0), RGD-hGVs (OD_500_ = 3.0), or PBS; scale bar, 200 μm. **(D–G)** Serum markers of hepatic function and renal function measured 7 days after injection of PBS, Con-hGVs, or RGD-hGVs (n = 3).

The hemolysis assay results in Figure 5A indicated that RGD-hGVs at various concentrations exhibited no hemolytic or agglutination effects on red blood cells. In contrast, Triton-X100-treated samples, serving as positive controls, demonstrated marked hemolysis. In contrast, the supernatant of RGD-hGV–treated samples remained clear and colorless after centrifugation, comparable to that of PBS-treated controls. In addition, RGD-hGVs did not differ from PBS with respect to hemolysis or agglutination, suggesting that RGD-hGVs did not cause damage to red blood cells. Incubation of K7M2, B16-F10, and bEnd.3 cells with different concentrations of RGD-hGVs followed by CCK8 assays reveared that RGD-hGVs exhibited no significant cytotoxicity to these cells (Figure 5B). H&E staining analysis revealed no pathological damage in the heart, liver, spleen, lungs, or kidneys, consistent with findings in the PBS-treated control group (Figure 5C). Additionally, *in vivo* biosafety was evaluated by examining blood samples collected from healthy mice after injection with RGD-hGVs or Con-hGVs. The data showed no significant abnormalities in blood biochemical indicators, including liver functions (ALT, AST) and kidney functions (BUN, CREA) (Figures 5D–G). These findings collectively suggest that RGD-hGVs exhibit favorable biosafety.

## 4 Discussion

Osteosarcoma and melanoma are among the most common malignant tumors (Tian et al., 2023; Siegel et al., 2024). The incidence of melanoma increases by 2%–3% annually.(Siegel et al., 2024). In their early stages, these tumors readily spread and metastasize through the bloodstream, causing severe prognosis for patients. Molecular imaging applications show substantial promise for the early diagnosis of these tumors (Hao et al., 2023). The αvβ3 integrin is highly expressed in numerous tumor cells, including osteosarcoma and melanoma cells (Liu et al., 2025; Zhong et al., 2017). It is closely associated with tumor cell invasion and metastasis and is also highly expressed in tumor vasculature. This discovery underscores the significance of αvβ3 integrin in osteosarcoma and melanoma, not only useful for imaging diagnosis but also for therapeutic interventions (Gu et al., 2022; Huilcaman et al., 2025; Zhu et al., 2023). In this work, we chose αvβ3 integrin as the molecular target for imaging and constructed an RGD-hGV acoustic probe for early detection of osteosarcoma and melanoma. The RGDfK peptide contains the Arg–Gly–Asp (R-G-D) motif and selectively recognizes αvβ3 integrin, which is abundantly expressed on tumor-associated vascular endothelial cells and on tumor cell membranes (Schottelius et al., 2009; Liu et al., 2021).

GVs from different microorganism sources exhibit variations in protein shell composition, geometric morphology, size distribution, and mechanical properties, which in turn influence their acoustic response characteristics and biological behavior (Pfeifer, 2012; Shapiro et al., 2014). Existing research indicates that hGVs generate stronger ultrasound contrast signals than eGVs at equivalent concentrations (Wang et al., 2025b; Yu et al., 2025). Therefore, in this study, we employed hGVs instead of the iRGD-modified sGVs we previously constructed to improve ultrasound molecular imaging performance. We selected two tumor cell lines with distinctly different origins and biological characteristics—K7M2 osteosarcoma and B16-F10 melanoma—to establish subcutaneous tumor models. Our results showed that RGD-hGVs significantly enhanced contrast signals in tumor regions in both models. While maintaining comparable peak signal intensities, these targeted hGVs exhibited markedly greater time-intensity curve (TIC) area and prolonged signal duration compared to non-targeted hGVs. Immunofluorescence staining analysis further confirmed the probe enriched in αvβ3-positive/CD31-positive regions. These findings demonstrate that the nanoscale αvβ3-targeted ultrasound molecular probe based on hGVs and the RGDfK peptide exhibits robust molecular imaging capabilities, not only in osteosarcoma but also in melanoma models, providing preliminary evidence for its broad applicability across different tumor types.

Clinical translation serves as a key metric for evaluating the potential value of novel contrast agents. This study demonstrated that RGD-hGVs exhibited low cytotoxicity to K7M2 and B16-F10 cells *in vitro*, with overall cell viability maintained above 90%. In healthy mice, no significant abnormalities were observed in serum liver and kidney function indicators after single intravenous injections at imaging doses and higher doses. Histological examination (H&E staining) of the major organs also revealed no apparent inflammatory responses or tissue damage. These findings align with previous safety evidence for GVs in myocardial infarction and liver disease models and further confirm the excellent biocompatibility of hGVs as a nanoscale ultrasound contrast agent scaffold (Wang et al., 2025b; Yu et al., 2025). RGD-hGVs leverage the high acoustic efficiency of hGVs to achieve strong imaging signal at lower dosages. Their biosynthesis and purification processes are relatively mature with high yields, facilitating scaled-up production and cost control. RGDfK has an established clinical foundation in nuclear medicine probes, enhancing predictability regarding its safety and *in vivo* metabolic behavior (Jia et al., 2026; Zang et al., 2022). PEG modification prolongs *in vivo* circulation and colloidal stability, while reducing non-specific uptake. All of these are helpful for subsequent clinical translation (Yu et al., 2025; Wang et al., 2020).

Several limitations remain in the present study. Firstly, this study has validated the ultrasound molecular imaging potential in only two tumor types, insufficient to cover all tumors exhibiting high αvβ3 expression. Factors such as the levels of αvβ3 expression, its spatial distribution (predominantly in vascular endothelium or tumor cells), tumor stroma density, and vascular permeability across various tumor types may significantly influence probe distribution and imaging efficacy. Moreover, the subcutaneous tumor models are not exactly the same as the tumor in clinical settings. Future studies should seek to validate the utility of RGD-hGVs in a wider spectrum of cancers, including breast cancer, non-small-cell lung cancer, and glioma.

## 5 Conclusion

In summary, we constructed a novel αvβ3-targeted ultrasonic molecular probe, RGD-hGVs. This probe is based on Halobacterium-derived gas vesicles. Our findings demonstrate that RGD-hGVs function as a new targeted probe with markedly improved imaging capability in melanoma and osteosarcoma. This improvement supports more accurate tumor diagnosis. RGD-hGVs show great promise as a broad-spectrum tumor-targeting nanoscale ultrasonic molecular probe for future clinical translation and application.

## Data Availability

The original contributions presented in the study are included in the article/Supplementary Material, further inquiries can be directed to the corresponding authors.
