# Supplementary material for: αvβ3-targeted gas vesicles for ultrasound molecular imaging of tumors
Source: Front Bioeng Biotechnol. 2026 Mar 27;14:1808539. doi: 10.3389/fbioe.2026.1808539 (PMC13066180; doi:10.3389/fbioe.2026.1808539)
Supplement: Supplementary file 1 [file Supplementaryfile1.docx]

**Supplementary materials
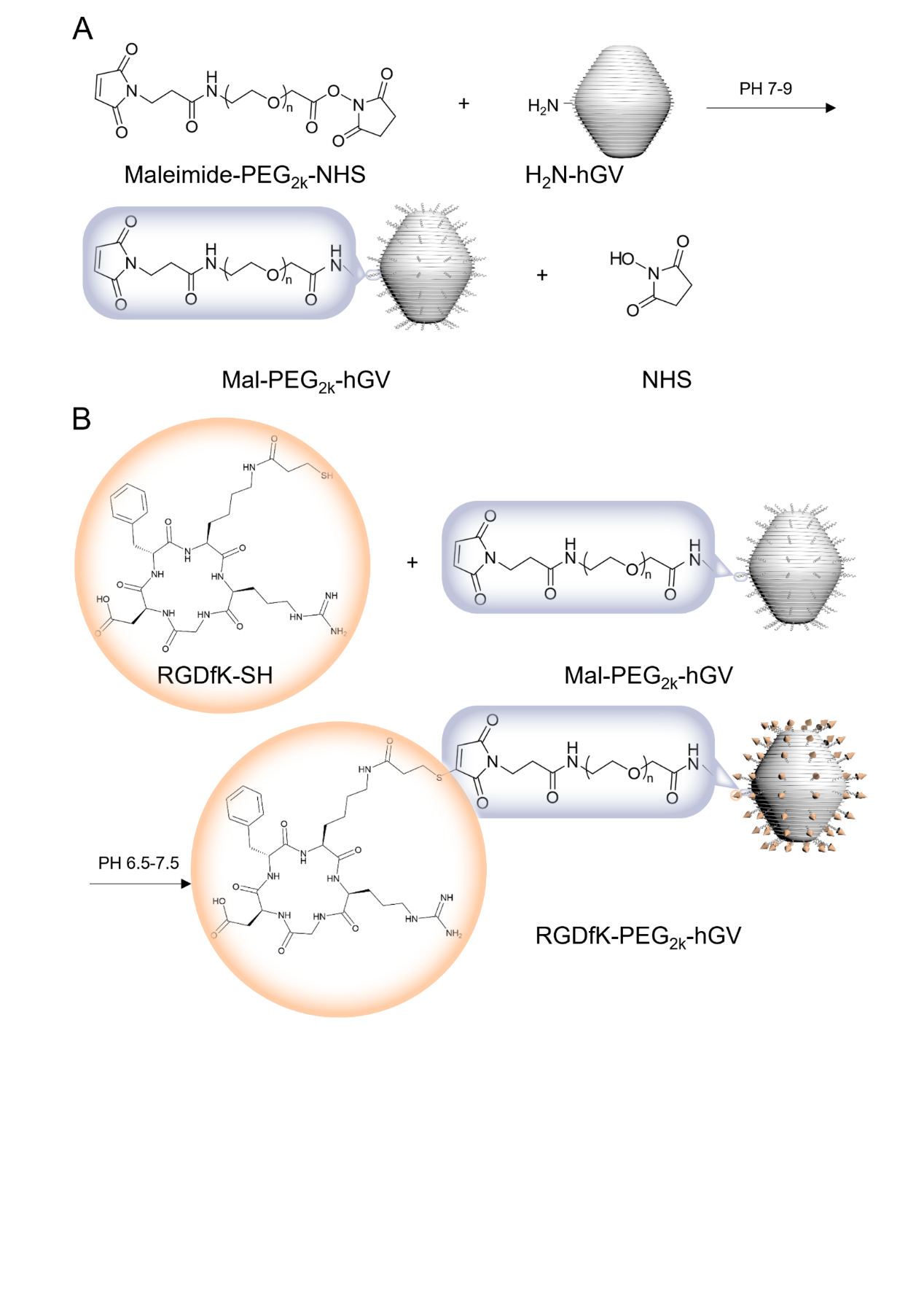
**

Figure S1｜Schematic of PEGylation and RGD conjugation of hGVs.

(A) Reaction of Mal-PEG_2k_-NHS with hGVs: The NHS active ester undergoes amidation with the −NH₂ groups on hGVs to form PEG-modified hGV (PEG-hGV); free NHS are removed by dialysis to form PEG-modified hGV (Mal-PEG_2k_-hGV); residual NHS are removed by dialysis.

(B) RGDfK coupling with PEG-hGVs: PEG terminal maleimide (Mal) undergoes addition reaction with sulfhydryl group (−SH) to form a thioether bond, yielding RGD-modified PEG-hGV (RGD-PEG-hGV).


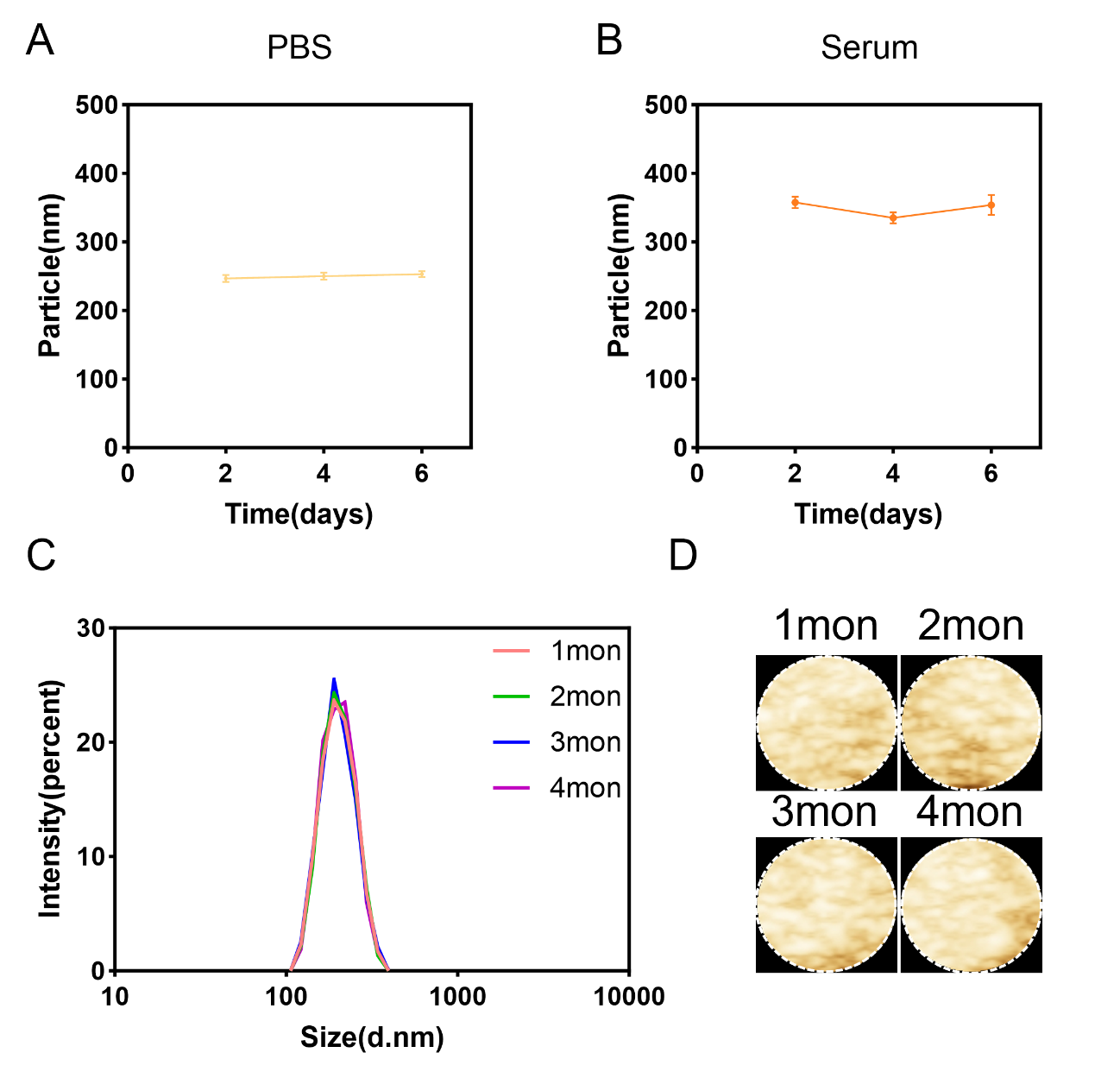


Figure S2｜Stability of PEG-hGVs in PBS/serum and storage stability of RGD-hGVs in PBS at 4℃.

(A) Hydrodynamic particle size of PEG-hGVs incubated in PBS as a function of time (days), measured by DLS. (B) Hydrodynamic particle size of PEG-hGVs incubated in serum as a function of time (days), measured by DLS. (C) DLS intensity-weighted size distributions of RGD-hGVs stored in PBS at 4℃ after 1, 2, 3, and 4 months. (D) Representative in vitro ultrasound contrast-mode images of RGD-hGVs stored in PBS at 4℃ after 1, 2, 3, and 4 months.


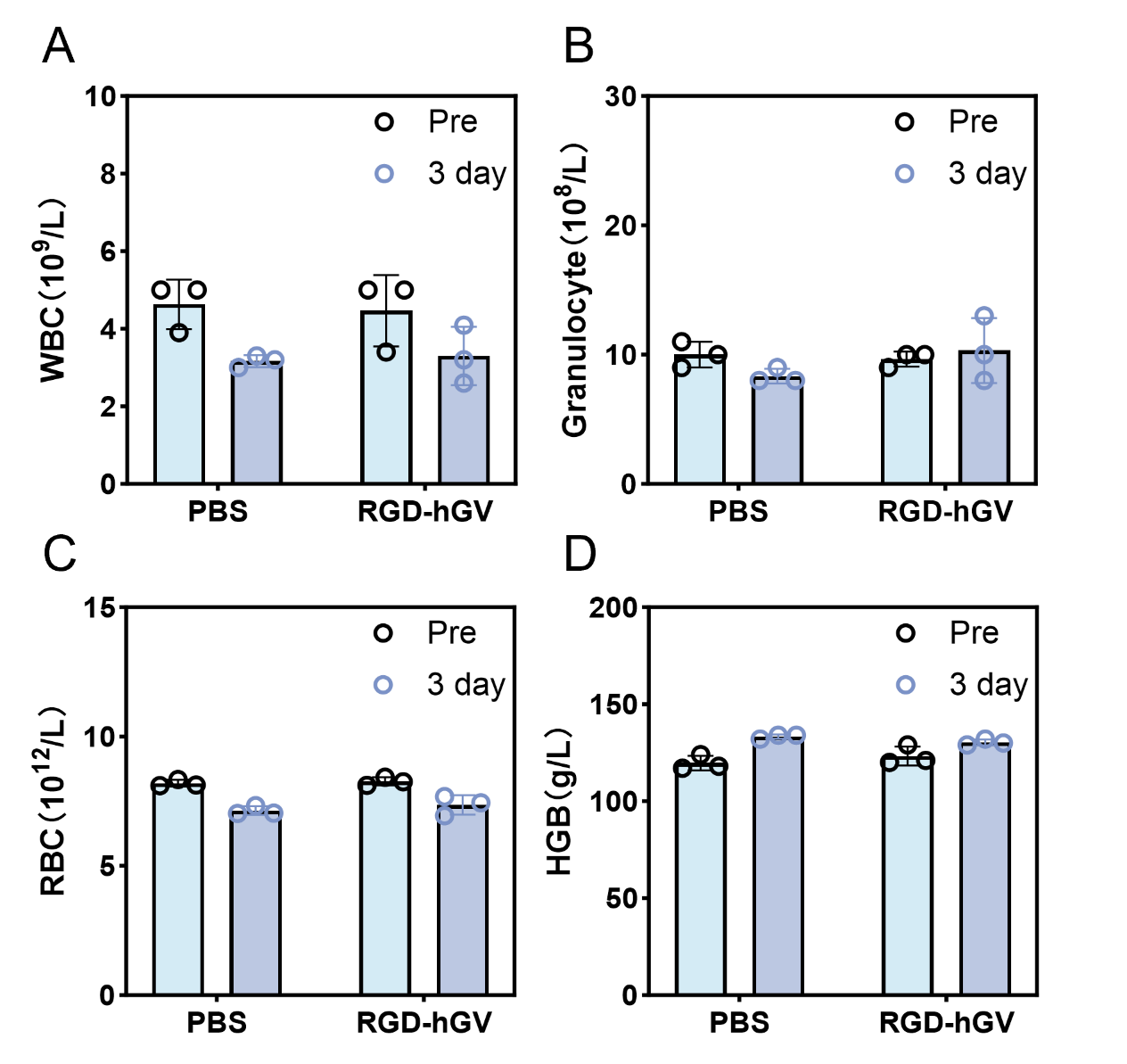


Figure S3｜Complete blood count (CBC) before and after injection.

CBC was measured pre-injection (Pre) and on day 3 post-injection (3 day) in animals receiving PBS or RGD-hGV. Shown are (A) white blood cell count (WBC), (B) granulocyte count, (C) red blood cell count (RBC), and (D) hemoglobin (HGB). No apparent abnormal changes were observed on day 3 compared with baseline, supporting systemic safety.


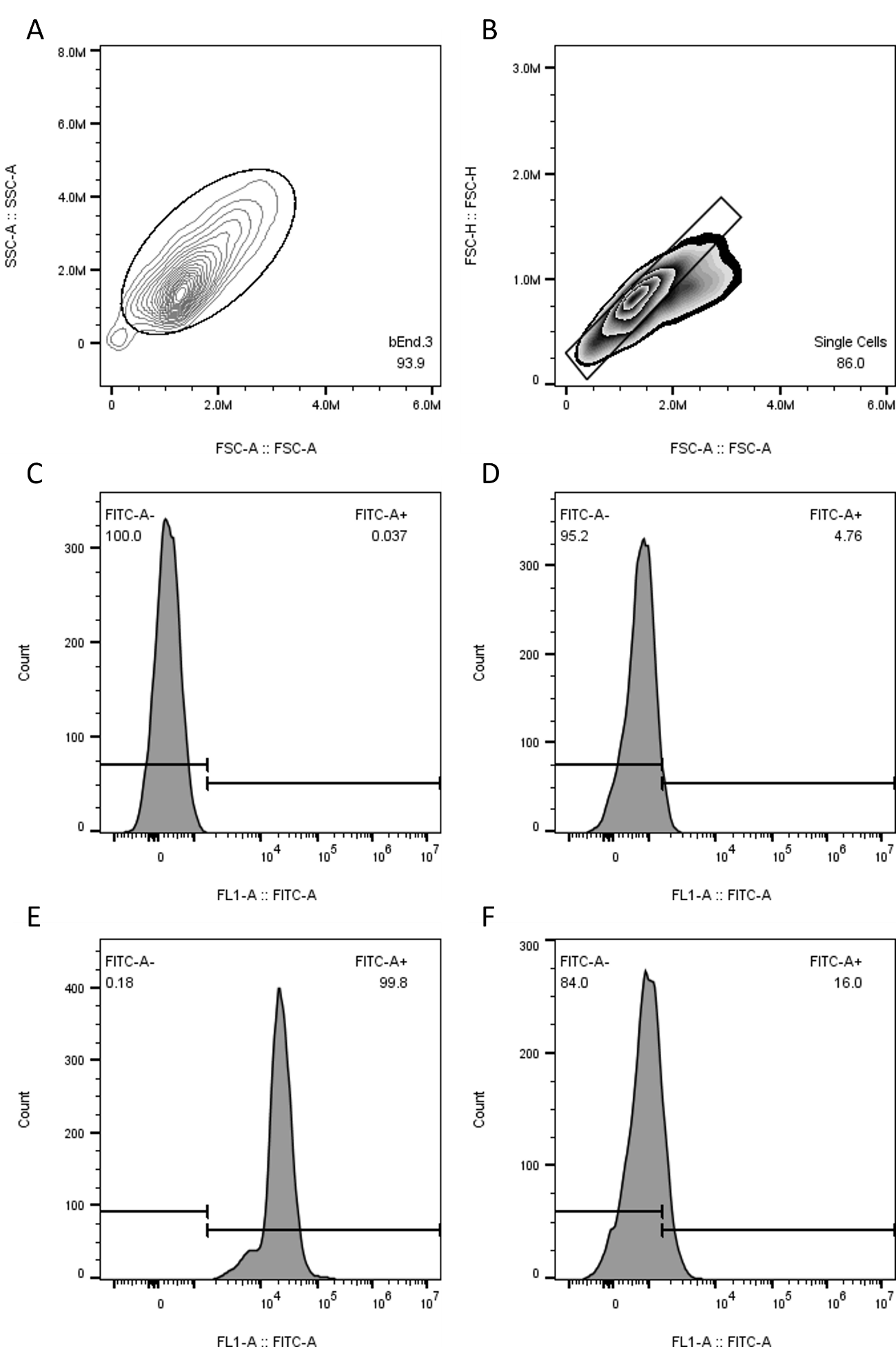


Figure S4｜Stepwise gating strategy and representative FITC histograms for bEnd.3 cells.
(A) FSC-A vs SSC-A for debris exclusion; (B) FSC-A vs FSC-H for singlet gating; (C) FITC-A histogram of unstained (blank) cells to define the FITC-positive threshold; (D–F) FITC-A histograms for (D) Control, (E) RGDfK-targeting, and (F) RGD-blocking (cells pre-incubated with free RGDfK) groups, applying the same FITC-positive threshold defined in (C) to all samples acquired under identical settings.


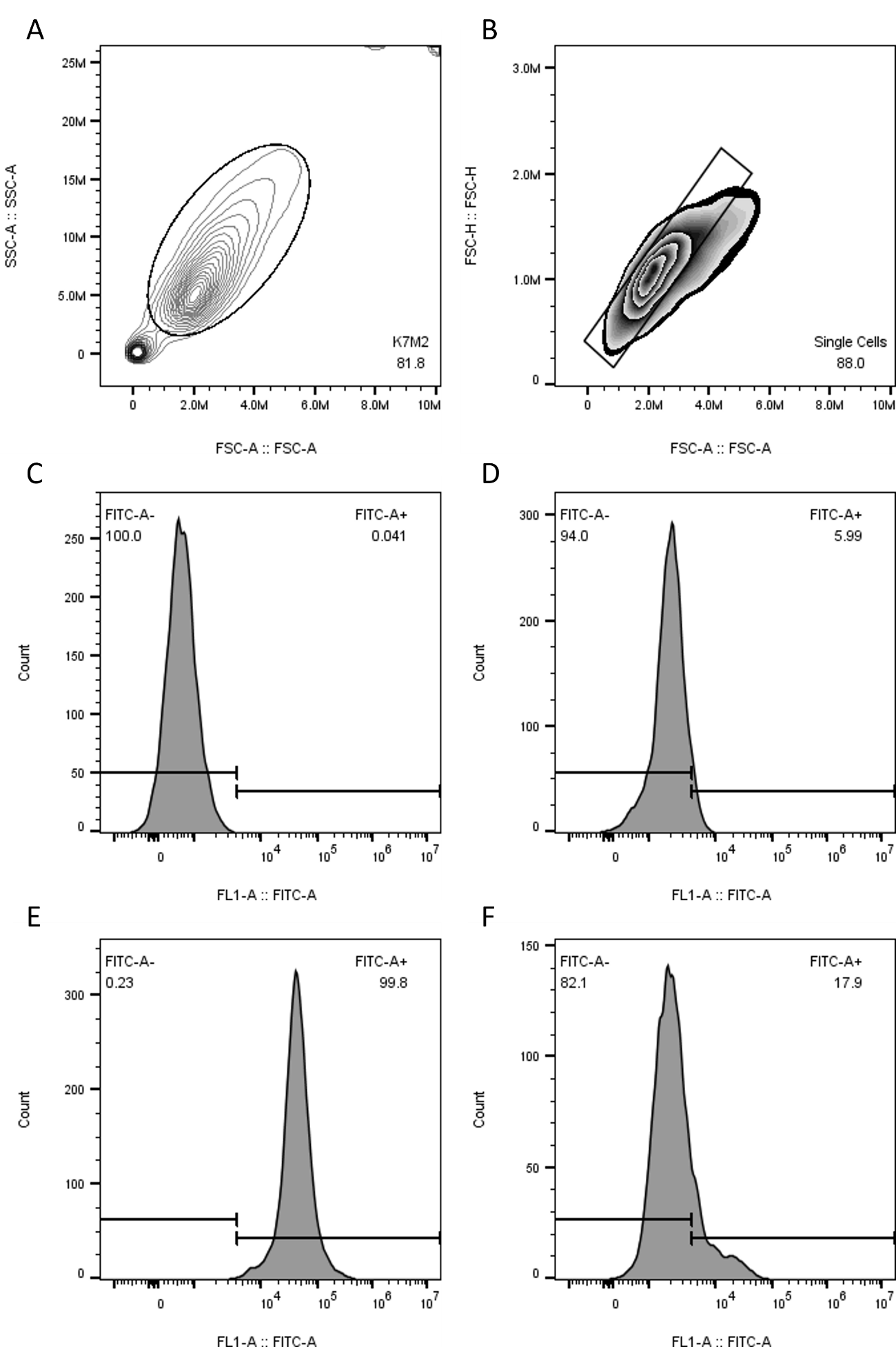


Figure S5｜Stepwise gating strategy and representative FITC histograms for K7M2 cells.
(A) FSC-A vs SSC-A for debris exclusion; (B) FSC-A vs FSC-H for singlet gating; (C) FITC-A histogram of unstained (blank) cells to define the FITC-positive threshold; (D–F) FITC-A histograms for (D) Control, (E) RGDfK-targeting, and (F) RGD-blocking (cells pre-incubated with free RGDfK) groups, applying the same FITC-positive threshold defined in (C) to all samples acquired under identical settings.


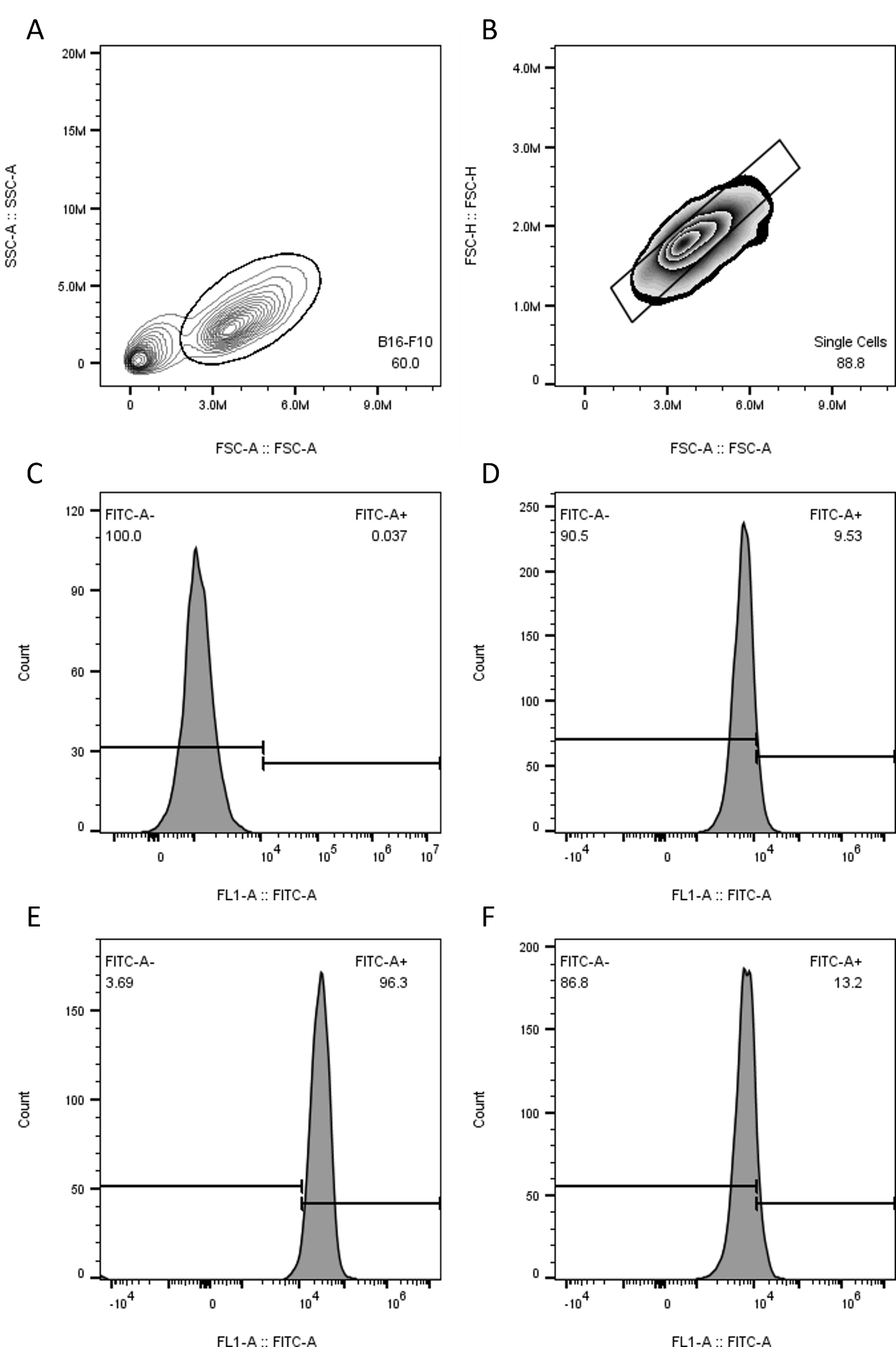


Figure S6｜Stepwise gating strategy and representative FITC histograms for B16-F10 cells.
(A) FSC-A vs SSC-A for debris exclusion; (B) FSC-A vs FSC-H for singlet gating; (C) FITC-A histogram of unstained (blank) cells to define the FITC-positive threshold; (D–F) FITC-A histograms for (D) Control, (E) RGDfK-targeting, and (F) RGD-blocking (cells pre-incubated with free RGDfK) groups, applying the same FITC-positive threshold defined in (C) to all samples acquired under identical settings.


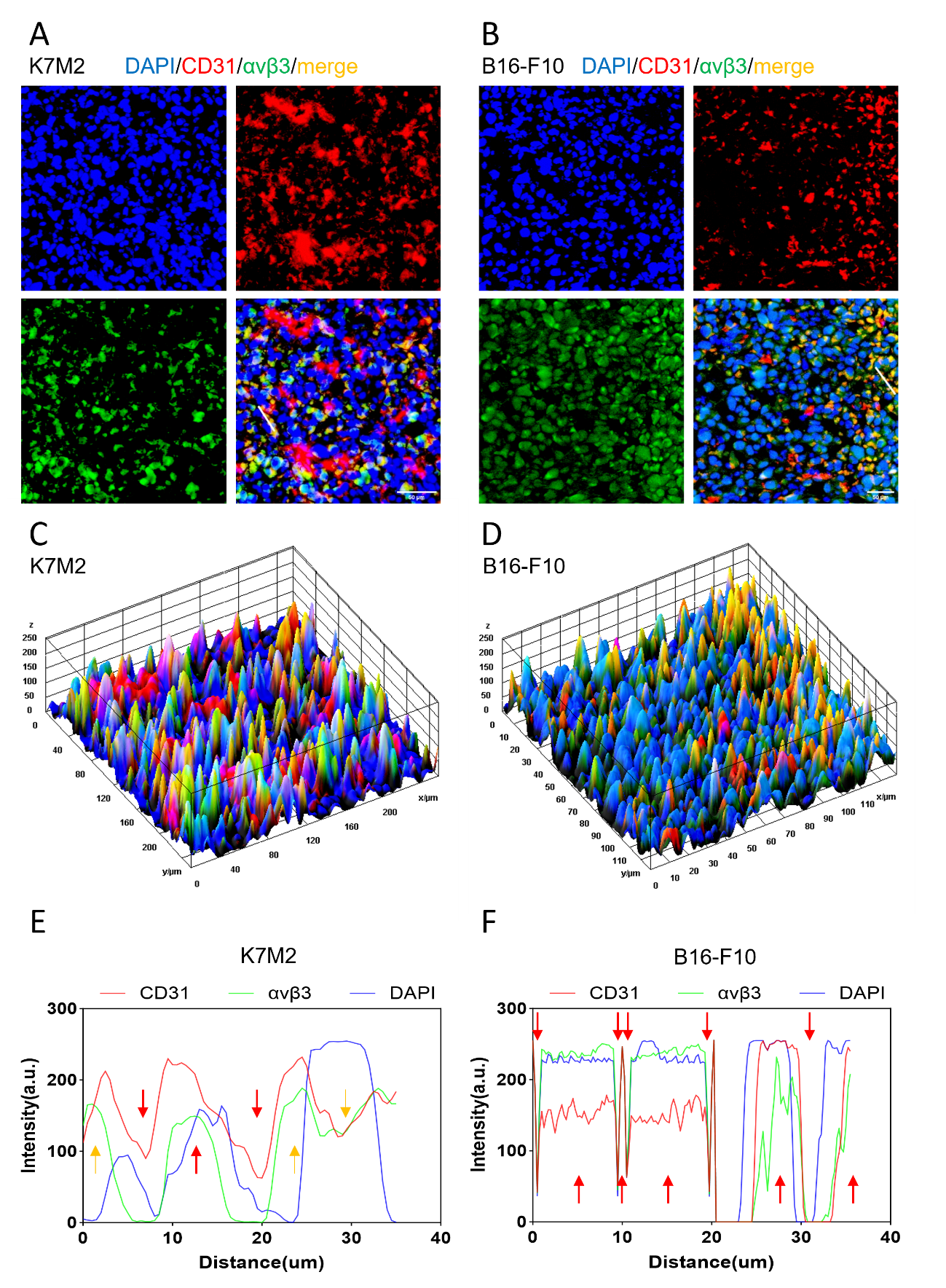
 Figure S7｜Immunofluorescence and colocalization analysis of K7M2 and B16-F10 tumor tissues.

(A, B) Immunofluorescence images of K7M2 osteosarcoma and B16-F10 melanoma, respectively; (C, D) corresponding 3D surface plots. (E, F) show enlarged co-localization images. The analysis regions are marked with white line in the merged images of (A) or (B). Yellow lines indicate CD31 co-localization with α_v_β_3_, while red arrows denote triple co-localization.

Table S1. Ultrasound imaging parameters.

| **Parameter** | **Abbreviation** | **Setting (Value)** |
| --- | --- | --- |
| Acoustic Power | AP | 5.13% |
| Mechanical Index | MI | 0.145 |
| Thermal Index (Soft tissue) | TIS | 0.004 |
| Frequency | F | CH7.1 |
| Depth | D | 2.5 |
| Gain | G | 70 |
| Frame Rate | FR | 12 |
| Dynamic Range | DR | 115 |

Table S2. Confocal microscopy acquisition settings.

| **Channel** | **Excitation (nm)** | **Emission window (nm)** | **Pinhole (AU)** | **HV** | **Offset** | **Detector gain** |
| --- | --- | --- | --- | --- | --- | --- |
| DAPI | 405 | 430–480 | 1.2 | 60 | 0 | 5.00 |
| FITC | 488 | 500–550 | 1.2 | 60 | 0 | 5.00 |
